# Climate-related drivers of nutrient inputs and food web structure in shallow Arctic lake ecosystems

**DOI:** 10.1038/s41598-022-06136-4

**Published:** 2022-02-08

**Authors:** Edoardo Calizza, Rosamaria Salvatori, David Rossi, Vittorio Pasquali, Giulio Careddu, Simona Sporta Caputi, Deborah Maccapan, Luca Santarelli, Pietro Montemurro, Loreto Rossi, Maria Letizia Costantini

**Affiliations:** 1 Department of Environmental Biology, Sapienza University of Rome, Via dei Sardi 70, 00185 Rome, Italy; 2 CoNISMa, National Inter-University Consortium for Marine Sciences, Rome, Italy; 3 ISP-CNR, Institute of Polar Sciences, National Research Council of Italy, Monterotondo, RM Italy; 4 IRSA-CNR, Water Research Institute, National Research Council, Monterotondo, RM Italy; 5 Department of Psychology, Sapienza University of Rome, Rome, Italy

**Keywords:** Ecology, Climate-change ecology, Community ecology, Ecological networks, Ecosystem ecology, Freshwater ecology, Stable isotope analysis, Wetlands ecology, Hydrology, Limnology

## Abstract

In order to predict the effects of climate change on polar ecosystems, disentangling mechanisms of nutrient transfer in food webs is crucial. We investigated sources of nutrients in tundra lakes, tracing their transfer through the food web and relating the observed patterns to runoff, snow coverage, and the presence of migratory geese in lake catchments. C and N content (elemental and isotopic) of several food web components including *Lepidurus arcticus* (Notostraca, at the top of the lake food webs) in 18 shallow Arctic lakes was compared. Terrestrial productivity and geese abundance were key biotic factors that interacted with abiotic variables (snow coverage, lake and catchment size) in determining the amount and origin of nutrient inputs, affecting the trophic interactions among aquatic species, food chain length and nutrient flow in Arctic lake food webs. Decreasing snow coverage, increasing abundance and expansion of the geese’s range are expected across the Arctic due to climate warming. By relating nutrient inputs and food web structure to snow coverage, vegetation and geese, this study contributes to our mechanistic understanding of the cascade effects of climate change in tundra ecosystems, and may help predict the response of lakes to changes in nutrient inputs at lower latitudes.

## Introduction

Global climate change is expected to affect nutrient cycling and transfer in food webs via physically and biologically mediated mechanisms^1–3^. Such changes will be particularly marked in the Arctic, due to the Arctic warming amplification^4–7^ and nutrient-limited conditions^8,9^. Admittedly, although abiotic changes are evidently transforming the region, our ability to predict their ecological effects is limited^10–12^.

Shallow Arctic lake ecosystems represent hotspots of biodiversity and productivity^6,13^ and provide ecosystem services both at the local and the global scale (e.g. they are important carbon sinks)^14,15^. Although they occupy less than 2% of the tundra, numerous aquatic and terrestrial species depend on these systems. Expected warming and greater precipitation will affect seasonal snow coverage, primary productivity, runoff in catchment areas and the density of migratory geese^16–19^, all of which represent important drivers of nutrient input and exchange between terrestrial and aquatic compartments^20,21^. Nevertheless, local-scale differences in these factors, as well as in lake and catchment area size, make it difficult to make general predictions about the effect of climate change on nutrient cycling and Arctic food webs.

A complete understanding of nutrient cycling in Arctic lake ecosystems should take account of food web-mediated effects on productivity and remineralisation processes^6,13,22,23^. Indeed, grazing by geese may affect vegetation biomass and the amount of nutrients stored in soil^24–28^, while top-down control by aquatic invertebrates may limit summer algal blooms even when high nutrient inputs are available^29^. In addition, consumers’ trophic niches are affected by resource availability and play an important role in nutrient transfer and food web structure^30–34^. Thus, they have the potential to modulate the effects of climate change and other stressors on ecosystems^22,35^.

In this context, C and N isotopic and elemental analyses represent powerful tools with which to quantify the relative importance of distinct sources of nutrients in sediments and soil, taken up by vegetation and transferred along food chains^36–39^. The isotopic signature of nitrogen (^15^N/^14^N, or δ^15^N, reported as per mil difference with respect to the atmospheric N_2_ standard, defining the Zero-Point of the nitrogen isotope scale) discriminates between organic and inorganic N inputs^40–43^, and can provide information on the trophic position of organisms in the food web^37,41,44–46^. Indeed, due to isotopic discrimination during metabolic reactions, the δ^15^N increases from a resource to its consumer. N of animal origin (e.g. goose feces) is thus enriched in ^15^N with respect to N originating from atmospheric N_2_ through fixation in soil or plants^44^. In parallel, the isotopic signature of carbon (^13^C/^12^C, or δ^13^C, reported as per mil difference with respect to the Vienna Pee Dee Belemnite standard, defining the Zero-Point of the carbon isotope scale) differs between terrestrial and aquatic vegetation, and makes it possible to disentangle the contribution of different C sources to aquatic food webs^36,38,47–50^. Indeed, differences in atmospheric and dissolved C sources, together with potential CO_2_ limitation in Arctic lakes which freeze solid during several months, produce enriched δ^13^C values in aquatic primary producers with respect to terrestrial ones^36,37,47^.

In this study, we investigated sources of nutrients and organic matter in tundra lake ecosystems, traced their transfer throughout the food web, and related the observed patterns to abiotic and biotic drivers that are likely to be influenced by climate change. To this end, we combined stable isotope analyses with remote sensing indices, Digital Elevation Models and information on migratory geese in 18 shallow Arctic lakes and their catchment areas (North Spitzbergen, Svalbard Islands). Isotopic and elemental C and N content in aquatic and terrestrial vegetation, plankton and benthic invertebrates, including the abundant species *Lepidurus arcticus* (Notostraca), as well as in the organic fraction of sediment and soil (hereafter referred to as ‘sediment’ and ‘soil’ respectively), were compared among lakes and related to differences in catchment area morphometry, snow coverage, vegetation (i.e. NDVI) and the amount of organic deposits (i.e. droppings) left by Barnacle geese (*Branta leucopsis*). *B. leucopsis* is the most abundant, the biggest and the only avian species that permanently occupies the study catchment areas during the short Arctic summer. It can thus be considered the animal species that contributes the most to nutrient inputs in these systems^17,29^. *L. arcticus* is the biggest species and it has no aquatic predators in our study lakes, and it dominates the relatively simple aquatic food web. In addition, it can adapt its diet to various food sources and it is a prey of certain bird species^51–53^. Hence, the two species represents the ideal candidates to study how variations in nutrient inputs potentially associated with climate change will affect food web structure and functioning.

We considered the effects that inputs of nutrients may produce by analysing the following questions: (i) Does vegetation in the catchment area, plausibly related to snow coverage^54^, affect the presence of geese and thus organic N inputs? (ii) Is terrestrial input in lakes, indicated by lower δ^13^C values in sediment, related to lake and catchment area size and thus to the potential runoff from the catchment area?

Based on optimal foraging theory^55,56^ and observations from temperate and Antarctic food webs^31,32,37,50,57^, we also hypothesised that a decrease in basal resource quality and quantity in lakes would be reflected in increased trophic generalism and omnivory among invertebrate species like *L. arcticus*. As a consequence, we expected cascade effects on food chain length and the coupling of distinct energy channels (i.e. detritivory and herbivory) in the food web^56,58^, two key properties affecting the stability of ecosystems^58,59^. Here, the ash free dry matter as a percentage (AFDM%) was considered to be a proxy for the overall resource quantity in lake sediment^31,57^, while the percentage of C in sediment was considered to be a proxy for resource quality, with a higher percentage being indicative of a lower quality. Indeed, while C is not limiting, a stoichiometric imbalance between C and other less abundant elements impairs consumer-resource interactions in food webs^60–62^. In relative terms, excess C is associated with nutrient limitation^60,62^ and, together with N, several nutrients including P and other trace elements that were not measured in this study have been found at limiting concentrations in the study lakes^29^.

These issues are not only of interest in Arctic lakes, since they also have the potential to improve predictions of lake ecosystems’ responses to climate-driven and human-induced changes in nutrient inputs at lower latitudes.

## Results

**Figure 1:**
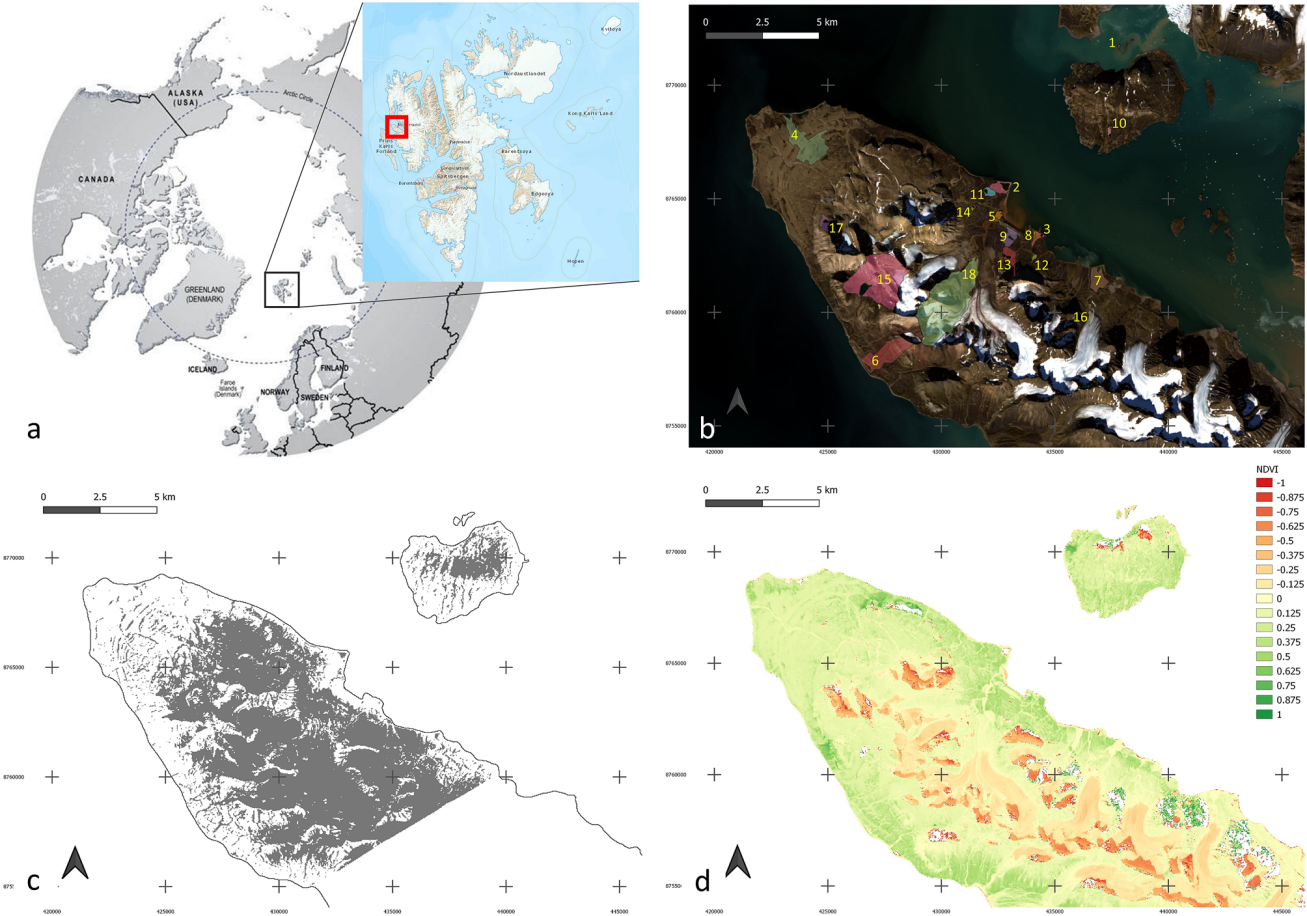
(**a**) Position of study area, the Brøgger peninsula (red square), in the Svalbard Islands in the Arctic circle. (**b**) Detail of the Brøgger peninsula showing the drainage basins of the study lakes (indicated by polygons of different colours to better differentiate basins, geographical coordinates provided in Table S2). Numbers indicate lakes ordered by distance from the coast. (**c**) Snow cover shown in grey in the study area at the beginning of the summer season (i.e. June). (**d**) Normalised Difference Vegetation Index (NDVI) in mid-August. The map of the Svalbard Islands in panel (**a**) and the background image in panel b were obtained from www.toposvalbard.com. Snow cover and NDVI were derived from Landsat 8 surface reflectance products, available on demand from the USGS (https://earthexplorer.usgs.gov/).

**Figure 2:**
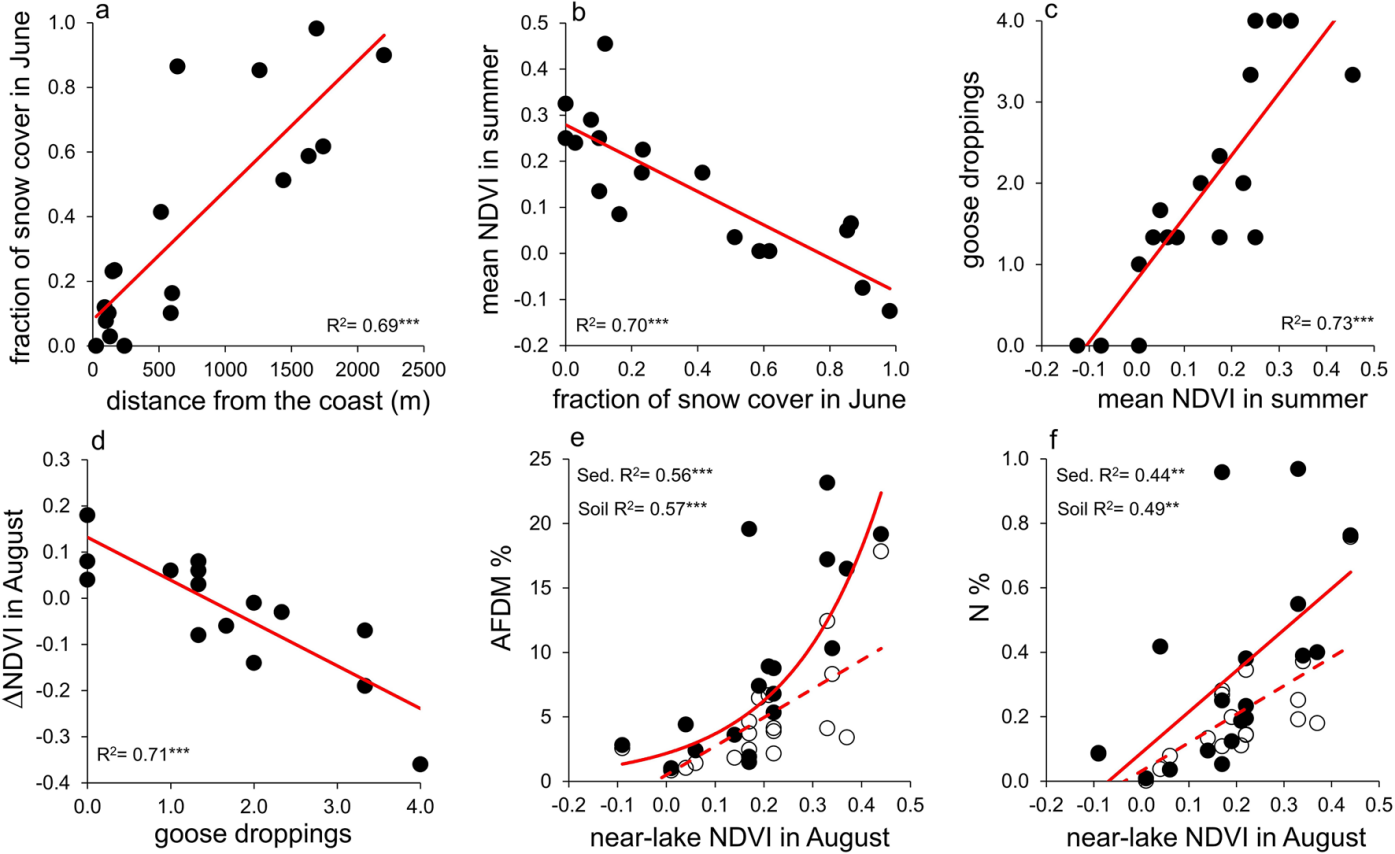
Correlations between abiotic and biotic variables measured in 18 shallow Arctic lakes. “Fraction of snow cover in June” refers to the fraction of each catchment area covered by snow at the beginning of summer. Mean NDVI in summer refers to the mean NDVI value from June to August. ΔNDVI refers to the difference between near-lake NDVI and catchment area NDVI in August (see "Methods" section). The relative amount of goose droppings, as a proxy for geese abundance, is expressed via non-integer values ranging from 0 (absent) to 4 (very high). Black and empty symbols in panels (**e**–**f**) indicate values in sediment and soil respectively. Asterisks denote the significance level: **: *p* < 0.01, ***: *p* < 0.001. See Appendix 1 in the online supplementary material for model details.

Lake and catchment surface areas were highly variable, respectively ranging from 0.002 Km^2^ and 0.010 Km^2^ to 0.160 Km^2^ and 5.350 Km^2^ (Fig. 1; Table S1). Openness ranged from 0.001 to 0.274 (Table 1). The snow coverage fraction (FSC_C_) varied markedly across catchment areas in June (Table 1 and Table S1), ranging from 0.00 to 0.98. Values increased with distance of the lakes from the coast (Fig. 2), and were negatively correlated with mean NDVI in summer (i.e. from June to August) (Fig. 2).

**Table 1:** Mean (± standard error) δ^13^C (‰), δ^15^N (‰), ash free dry matter (AFDM, %) and N content (%) in the sediments of 18 shallow Arctic lakes and soil in their catchment areas in North Spitzbergen (Svalbard Islands).

Lake	Dist	L/C	FSC_C_	NDVI	Droppings	Sediment				Soil			
						δ^13^C	δ^15^N	AFDM	N	δ^13^C	δ^15^N	AFDM	N
1-M	25	0.18	0.00	0.33	4.0 ± 0.0	− 22.2 ± 0.5	8.2 ± 0.4	4.4 ± 0.4	0.42 ± 0.03	− 24.5 ± 0.4	2.2 ± 0.9	1.0 ± 0.2	0.04 ± 0.02
2-S	90	0.24	0.12	0.46	3.3 ± 0.3	− 22.2 ± 0.4	3.7 ± 0.3	1.9 ± 0.3	0.05 ± 0.01	− 25.4 ± 0.3	2.2 ± 0.6	4.6 ± 1.1	0.28 ± 0.11
3-M	100	0.07	0.08	0.29	4.0 ± 0.0	− 24.3 ± 0.3	5.5 ± 0.7	19.2 ± 4.0	0.76 ± 0.13	− 25.7 ± 0.2	4.6 ± 1.3	17.8 ± 5.6	0.76 ± 0.30
4-M	120	0.02	0.10	0.14	2.0 ± 0.0	− 17.1 ± 0.2	2.1 ± 0.1	19.6 ± 4.6	0.96 ± 0.34	− 25.8 ± 0.2	3.9 ± 0.3	3.7 ± 1.6	0.27 ± 0.11
5-S	130	0.05	0.03	0.24	2.3 ± 0.3	− 24.8 ± 0.3	3.5 ± 0.4	6.8 ± 0.5	0.19 ± 0.03	− 25.7 ± 0.2	3.0 ± 0.5	4.1 ± 1.0	0.34 ± 0.16
6-S	150	0.08	0.23	0.18	2.0 ± 0.0	− 20.9 ± 0.2	3.3 ± 0.1	5.3 ± 0.7	0.23 ± 0.03	− 25.6 ± 0.4	2.4 ± 0.6	2.2 ± 0.6	0.14 ± 0.04
7-M	165	0.05	0.23	0.23	3.3 ± 0.3	− 22.6 ± 0.4	3.2 ± 0.3	1.5 ± 0.1	0.25 ± 0.01	− 24.9 ± 0.3	1.7 ± 0.6	2.5 ± 1.0	0.11 ± 0.04
8-M	240	0.07	0.00	0.25	4.0 ± 0.0	− 25.7 ± 0.2	4.1 ± 0.4	17.2 ± 1.9	0.55 ± 0.12	− 25.5 ± 0.1	4.0 ± 1.0	12.4 ± 1.4	0.25 ± 0.06
9-L	515	0.06	0.41	0.18	1.3 ± 0.3	− 23.5 ± 0.5	1.4 ± 0.3	10.3 ± 2.9	0.39 ± 0.11	− 25.9 ± 0.1	2.7 ± 0.3	8.3 ± 2.0	0.37 ± 0.11
10-L	590	0.24	0.10	0.25	1.3 ± 0.3	− 17.9 ± 0.3	1.7 ± 0.4	23.2 ± 7.7	0.97 ± 0.25	− 25.0 ± 0.3	3.0 ± 0.2	4.1 ± 0.6	0.19 ± 0.04
11-L	600	0.09	0.16	0.09	1.3 ± 0.3	− 24.6 ± 0.2	2.6 ± 0.3	16.5 ± 6.0	0.40 ± 0.07	− 25.6 ± 0.1	2.5 ± 0.5	3.4 ± 0.6	0.18 ± 0.03
12-L	640	0.04	0.86	0.07	1.3 ± 0.3	− 25.0 ± 0.1	4.0 ± 1.1	8.9 ± 1.4	0.19 ± 0.03	− 25.1 ± 0.3	1.6 ± 0.2	6.7 ± 1.5	0.11 ± 0.03
13-L	1260	0.11	0.85	0.05	1.7 ± 0.3	− 25.8 ± 0.1	2.2 ± 0.1	7.4 ± 1.7	0.12 ± 0.02	− 25.9 ± 0.7	2.4 ± 0.4	6.5 ± 1.7	0.20 ± 0.05
14-L	1440	0.27	0.51	0.04	1.0 ± 0.0	− 19.7 ± 1.2	3.8 ± 1.2	8.8 ± 3.7	0.38 ± 0.12	− 25.2 ± 0.2	2.7 ± 0.2	3.9 ± 0.5	0.14 ± 0.02
15-G	1630	0.04	0.59	0.01	2.0 ± 0.0	− 22.9 ± 0.2	2.5 ± 0.3	2.8 ± 0.4	0.08 ± 0.02	− 24.7 ± 0.3	3.1 ± 0.4	2.6 ± 0.4	0.09 ± 0.02
16-G	1690	0.19	0.98	− 0.13	0.0 ± 0.0	− 22.7 ± 0.2	2.1 ± 0.4	1.0 ± 0.1	0.01 ± 0.00	− 22.8 ± 0.3	− 0.9 ± 0.6	0.8 ± 0.2	0.01 ± 0.00
17-G	1740	0.14	0.62	0.01	0.0 ± 0.0	− 24.6 ± 0.9	1.2 ± 1.1	3.6 ± 0.8	0.09 ± 0.03	− 25.9 ± 0.1	2.1 ± 0.6	1.8 ± 0.1	0.13 ± 0.02
18-G	2200	0.01	0.90	− 0.08	0.0 ± 0.0	− 24.8 ± 0.1	0.4 ± 0.5	2.4 ± 0.2	0.04 ± 0.00	− 25.1 ± 0.2	1.2 ± 0.6	1.4 ± 0.1	0.09 ± 0.01

“Dist.”: distance from the centre of the lake to the coast (m), “L/C”: ratio of lake to catchment surface area, “FSC_C_”: fraction of snow coverage in the catchment area at the beginning of summer (June), “NDVI”: mean NDVI during summer (i.e. from June to August), “Droppings”: relative amount of goose droppings, ranging from 0 (absent) to 4 (very high). Lakes are numbered according to increasing distance from the coast and capital letters indicate lake category: M: “*muddy coastal*”; S: “*sandy coastal*”; L: “*lowland*”; G: “*glacier*”.

**Figure 3:**
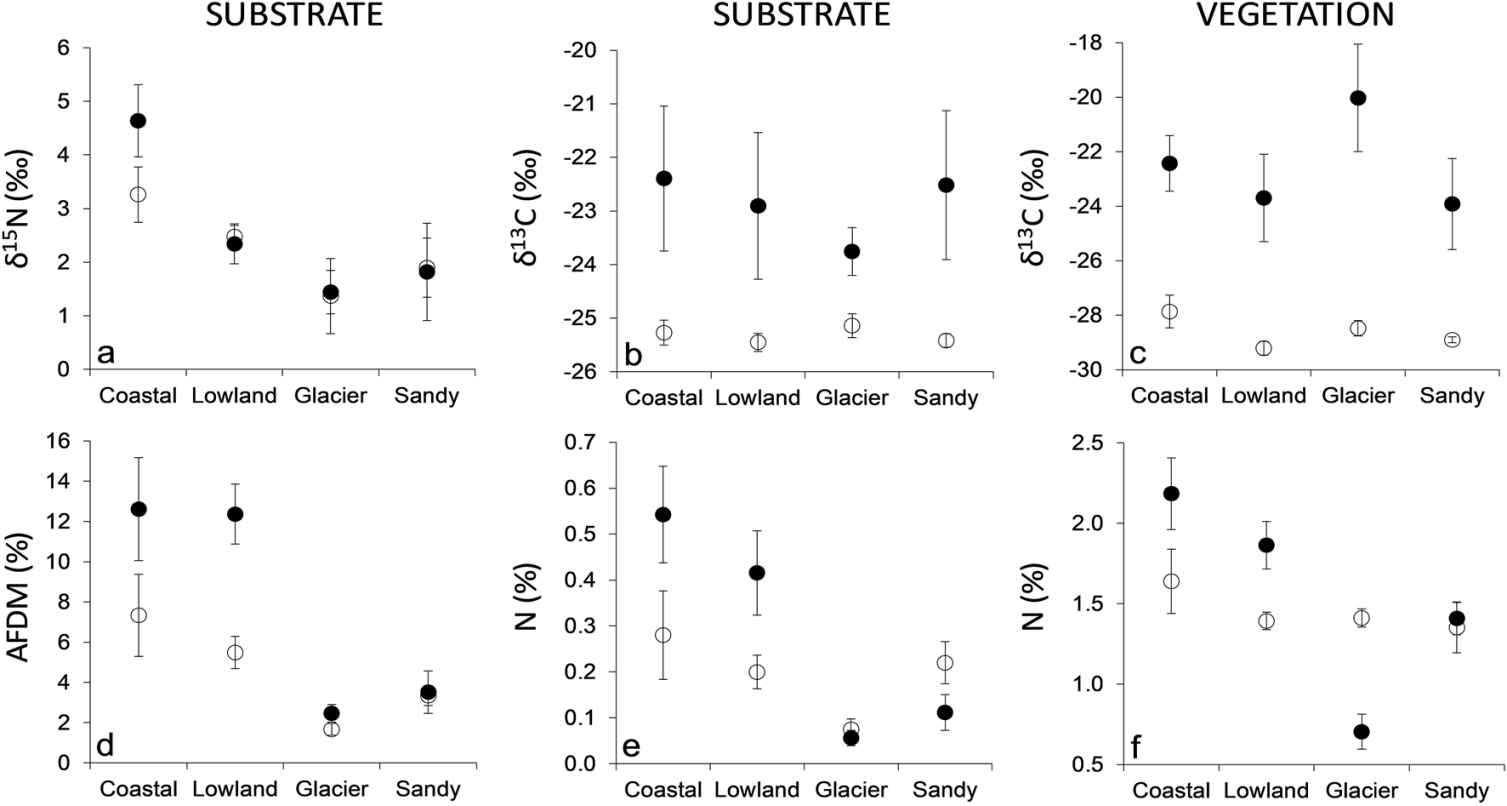
δ^15^N, δ^13^C, ash free dry matter as % (AFDM) and N content as % in various lake categories in North Spitzbergen (Svalbard Islands). “Coastal” refers to “*Muddy coastal*” lakes, while “Sandy” refers to “*Sandy coastal*” lakes. “SUBSTRATE” refers to the comparison of sediment (black symbols) and soil (empty symbols). “VEGETATION” refers to the comparison of aquatic vegetation (black symbols) and terrestrial vegetation (empty symbols). Statistics are reported in Table 2.

The isotopic C and N values of sediment and soil varied across lakes and across catchment areas (Table 1; one-way PERMANOVA, sediment: F = 17.6. *p* < 0.0001; soil: F = 4.4. *p* < 0.0001), but the differences were not related to the distance between them (Mantel test, Sediment: R = − 0.04, *p* > 0.05; Soil: R = − 0.07, *p* > 0.05). Mean δ^13^C values in sediment, soil and benthic and terrestrial vegetation did not vary significantly among lake categories but were enriched in lakes (i.e. in sediment and benthic vegetation) than in their catchment areas (i.e. in soil and terrestrial vegetation respectively) (Table 2 and Fig. 3). In contrast, δ^15^N, N% and AFDM% values in sediment and soil were generally higher in “*Muddy coastal*” lakes and lower in “*Glacier*” and “*Sandy coastal*” lakes (Table 2 and Fig. 3).

**Table 2:** Two-way ANOVA testing the effect of lake category (i.e. “*Muddy costal*”, “*Sandy coastal*”, “*Lowland*” and “*Glacier*”), ecosystem compartment (i.e. aquatic vs. terrestrial) and their interaction on δ^15^N, δ^13^C, AFDM and N content in the substrate, S. (i.e. sediment/soil), as well as on δ^13^C and N content in aquatic and terrestrial vegetation, V., in shallow Arctic lakes in North Spitzbergen (Svalbard Islands).

Variable	Factor	F	*p* value
S. δ^15^N (‰)	Compartment	0.30	0.587
	Category	4.66	**0.008**
	Interaction	0.53	0.67
S. δ^13^C (‰)	Compartment	12.14	**0.001**
	Category	0.17	0.918
	Interaction	0.22	0.880
S. N (%)	Compartment	4.32	**0.049**
	Category	4.75	**0.008**
	Interaction	1.68	0.191
S. AFDM (%)	Compartment	4.69	**0.038**
	Category	5.86	**0.003**
	Interaction	1.16	0.341
V. δ^13^C (‰)	Compartment	14.26	**0.000**
	Category	0.33	0.804
	Interaction	0.32	0.813
V. N (%)	Compartment	3.62	0.061
	Category	8.31	**0.000**
	Interaction	3.17	**0.029**

Bold values indicate a significant effect, *p* < 0.05. Statistics refer to values shown in Fig. 3.

The abundance of goose droppings increased with mean NDVI in summer (Fig. 2), while it was negatively correlated with ΔNDVI in August (Fig. 2). Also, from June to August, near-lake NDVI decreased most around those lakes where goose droppings were most abundant (Appendix 1, R^2^ = 0.33, *p* = 0.01). In turn, near-lake NDVI in August was positively correlated with AFDM% and N% in both soil and sediment (Fig. 2), which were not related to catchment area NDVI, regardless of the month considered (R^2^ always < 0.21, *p* always n.s.).

### Nutrient inputs and lake food webs

**Figure 4:**
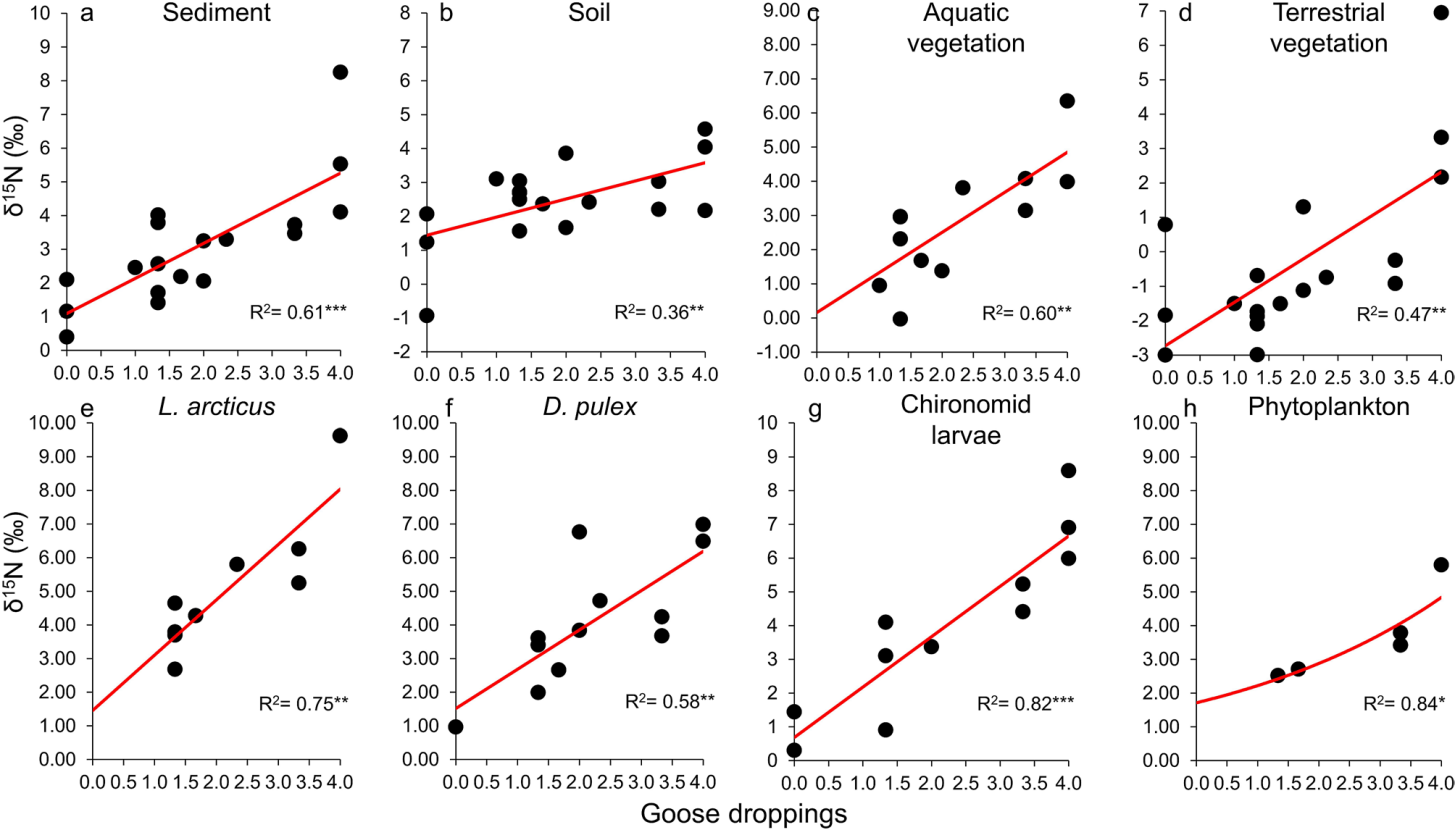
Correlations between the relative amount of goose droppings, as a proxy for geese abundance, and δ^15^N in various aquatic and terrestrial food web components in 18 shallow Arctic lakes and their catchment areas, North Spitzbergen (Svalbard Islands). *L. arcticus*: *Lepidurus arcticus* (Notostraca). *D. pulex*: *Daphnia pulex* (Cladocera). The relative amount of goose droppings is expressed via non-integer values ranging from 0 (absent) to 4 (very high). Each dot represents one lake ecosystem. Not all components were found in all lakes (see Table S2 for details). The maximum and minimum values on the vertical axes are selected in order to show the same δ^15^N range (i.e. a span of 10‰). Asterisks denote the significance level: *: *p* < 0.05, **: *p* < 0.01, ***: *p* < 0.001. See Appendix 1 in the online supplementary material for model details.

The δ^15^N values of the terrestrial and aquatic food web components analysed in this study increased with the abundance of goose droppings (Fig. 4 and Appendix 1), whose δ^15^N ranged between 4.3 ± 0.4‰ (Lake 5) and 7.8 ± 2.8‰ (Lake 3). The N% of terrestrial vegetation increased with its δ^15^N (Appendix 1, R^2^ = 0.66, *p* < 0.001), while no significant relationships were found for the N% of aquatic vegetation. Sediment δ^13^C varied with neither the abundance of goose droppings nor NDVI, regardless of the month and spatial scale considered (R^2^ always < 0.10, *p* always n.s.), while it decreased with lake openness (Appendix 1, R^2^ = 0.45, *p* < 0.01).

*L. arcticus* was found in 9 out of 18 lakes (Table S2). It appeared to be more frequent in lakes with benthic vegetation than lakes without (Table S2, χ^2^ test, χ^2^ = 9.0, *p* < 0.01), while no differences in mean distance from the coast, goose droppings, AFDM%, N% and sediment δ^13^C and δ^15^N were found between lakes with and without *L. arcticus* (t-tests, t always < 1.3, *p* always n.s.).

**Figure 5:**
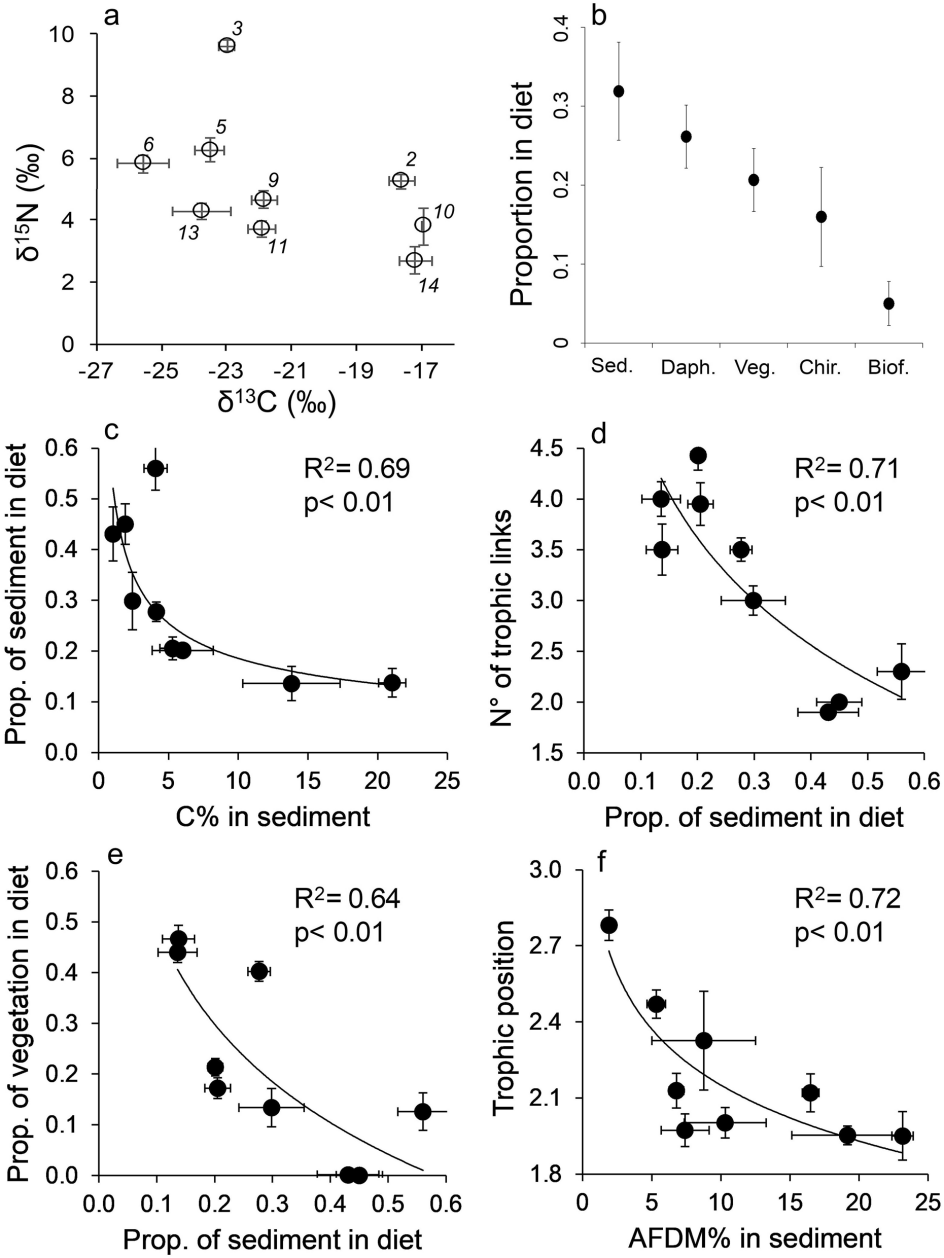
(**a**) Mean (± standard error among individuals) δ^13^C and δ^15^N values in *Lepidurus arcticus* (Notostraca). Numbers close to symbols indicate the lake number as reported in Table 1. (**b**) Mean (± standard error among lakes) proportional contribution of various food sources to the diet of *L. arcticus*, obtained through individual Bayesian isotopic mixing models (MixSIAR in R software). “Sed”: sediment, “Daph.”: *Daphnia pulex*, “Veg.”: benthic vegetation, “Chir.”: chironomid larvae, “Biof.”: biofilm. (**c**, **d**) Correlations between C and ash free dry matter content (AFDM), as %, in lake sediment and the trophic niche and position of *Lepidurus arcticus*. “N° of trophic links” refers to the number of distinct food sources that contributed to the diet of *L. arcticus* within each lake.

The isotopic signatures of *L. arcticus* varied between lakes (Fig. 5, one-way PERMANOVA, F = 138.9, *p* < 0.0001). Overall, sediment, *Daphnia pulex* and benthic vegetation contributed most to its diet (Fig. 5, one-way ANOVA, F = 3.2, *p* < 0.05), and sediment turned out to be higher quality food than aquatic vegetation (i.e. it had a lower C:N ratio; C/N in sediment = 14.7 ± 1.9 vs. C/N in aquatic vegetation = 21.1 ± 1.6, paired t-test, t = 3.7, *p* < 0.01). The contribution of sediment to *L. arcticus*’ diet increased as C content (C%) decreased. When such contribution increased, that of benthic vegetation and the number of resources in *L. arcticus*’ diet decreased (Fig. 5). Lastly, the trophic position of *L. arcticus*, which determined the maximum food chain length in lake food webs, was positively correlated with the contribution of *D. pulex* to its diet (R^2^ = 0.67, *p* < 0.01), while it was negatively correlated with AFDM% in sediment (Fig. 5). The isotopic signatures of *L. arcticus* and its potential food sources can be found in Table S3.

## Discussion

In order to predict the effects of climate change on biodiversity and functioning in Arctic ecosystems, disentangling the mechanisms of nutrient transfer in food webs is crucial. Indeed, in these systems, climate is warming faster than any other place on Earth and nutrients severely limit biological productivity^4,8^. Here, the exceptional natural laboratory of the Brøgger peninsula, in the North-Western sector of Svalbard, allowed us to compare shallow lakes differing markedly in their morphology, seasonal snow coverage and the presence of migratory geese in their catchment areas, despite being relatively close to each other. Multispectral satellite images and the integrated analysis of snow and vegetation indexes proved useful for studying the effects of seasonal climatic variations on tundra lake ecosystems, as previously shown in the Arctic^63–70^. In addition, the absence of any association between the geographical distance between lakes and the isotopic composition of sediment and soil suggests that potential local-scale differences in geology did not affect the observed results.

Snow coverage in June predicted the mean NDVI during summer (i.e. from June to August), which in turn was a strong predictor of the presence of geese across the landscape. Geese then explained local-scale differences between catchment-area and near-lake NDVI, the latter being relatively lower around lakes where their presence was higher. Notably, near-lake NDVI predicted the amount of organic matter and nitrogen stored in both soil and sediment, which significantly affected the length of the food chain and the number of food sources consumed by *L. arcticus*, a key species in lake food webs and the prey of other birds.

**Figure 6:**
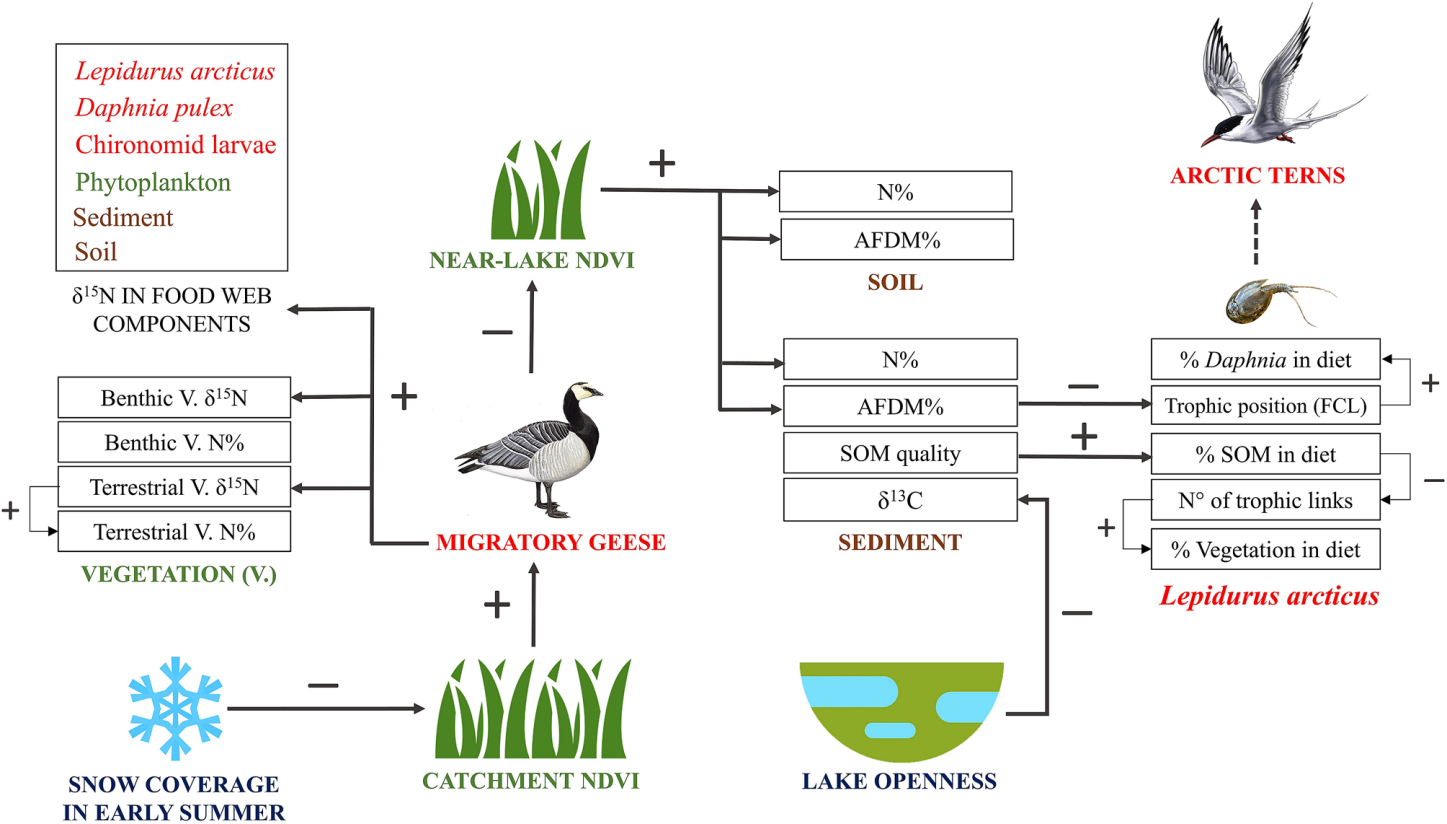
Schematic model showing the effects of abiotic factors (i.e. snow coverage and lake openness) and biotic factors (i.e. migratory geese and NDVI) on nutrient inputs and food webs in tundra ecosystems, as determined through the study of 18 shallow Arctic lakes and their catchment areas (Svalbard Islands). Colours indicate food web components, brown: substrate, green: primary producers, red: consumers. + and − indicate a significant positive and negative effect respectively (see Appendix 1 for model details). “Lake openness” is calculated as the lake-to-catchment surface area ratio. The higher this ratio, the lower the openness. Effects on the trophic niche and position of *Lepidurus arcticus* (Notostraca), a key species in the food web and the prey of other bird species, are shown. The trophic position of *L. arcticus* determined the maximum food chain length (FCL), as well the nutrient flow through the detritus or herbivore pathways in lake food webs. The dashed arrow indicates that Arctic Terns (*Sterna paradisaea*) were frequently observed preying on *L. arcticus* during this study, but this trophic link was not quantified. AFDM% and N%: ash free dry matter and N content respectively. SOM: sediment organic matter. δ^15^N (‰) and δ^13^C (‰): N and C stable isotope values respectively. Higher δ^15^N indicates increasing goose-derived N inputs. Lower δ^13^C in lake sediment indicates increasing terrestrial C inputs.

Our results are consistent with local-scale manipulative field experiments, which demonstrated that grazing by geese reduced plant litter and thus the amount of organic matter and nutrients in soil^25–27^, although they also suggest that terrestrial vegetation affects nutrient deposition in lake sediments. In addition, the onset of spring and vegetation productivity have been found to be closely associated with snow coverage in Arctic ecosystems^54^, as observed across the landscape in our study. Seasonal snow coverage and goose populations will be strongly affected by climate change, with reduced snow, increased geese abundance and northward range expansion expected across the Arctic^17^. Thus, by relating nutrient inputs and food web structure to snow coverage, vegetation and geese distribution, the results of this study contribute to our mechanistic understanding of climate change’s cascade effects on tundra ecosystems (Fig. 6).

The isotopic approach allowed us to observe landscape-scale patterns in terms of the organic vs. inorganic origin of N inputs, as well as the importance of aquatic vs. terrestrial C inputs in lake sediment. Based on differences in the presence of a key species (the Barnacle goose) and morphology (i.e. lake and catchment surface area), we were able to compare lakes spanning a broad range of sizes and distances from the coast within a coherent framework. Our data suggest that the warming and lengthening of the ice-free season, which promote aquatic productivity^17,52^, drive ecological changes in lakes with low openness and far from the coast, where nutrients were mainly of autochthonous origin. In contrast, “Arctic greening” and warmer winters along birds’ migratory routes^71^, together with increasing precipitation^17^, are expected to drive changes in lakes close to the coast with high openness, where organic inputs were mainly related to geese (for N) and runoff of terrestrial material (for C).

As shown by others^29,72–76^ the quantification of goose droppings, together with the characterisation of various food web components, proved to be an effective way of evaluating the role of geese on N inputs, demonstrating their pervasive effect on lake ecosystems. As expected^77^, sandy substrates in coastal lakes retained little nitrogen and organic matter, showing values similar to lakes close to glaciers, where geese were absent and primary production was low. This suggests an interaction between geese and substrate that should be considered in future characterisations of geese breeding grounds.

Geese affected the nitrogen stored in soil, sediment and vegetation. While their grazing activity may counterbalance the positive effect of N inputs on grass vegetation (the geese’ preferred food source) at high grazing pressure^24,27^, such inputs may have net positive effects on the remaining food web components. Indeed, increased moss coverage and N uptake have been shown to be related to geese inputs^29,78^, and higher biomass and diversity of phytoplankton and invertebrates have been shown to be related to goose-mediated nutrient enrichment in lakes^76^, while higher N concentrations increase vegetation productivity and its nutritional value for other Arctic herbivores^79,80^. In addition, Arctic terns were observed preying on *L. arcticus* during this study. Data indicated that organic N inputs from geese affected the δ^15^N values of *L. arcticus*, thus suggesting a transfer of goose-derived nutrients to predatory bird species.

Bird-mediated fertilisation of summer grounds may provide an advantage to migratory species that show philopatry, particularly at low population densities when intraspecific limitation and grazing pressure are weak^71,81,82^. With reference to geese, the higher the quality and productivity of vegetation, the higher the probability of successful fledging and the greater the accumulation of bodily reserves for migration to wintering grounds^71^. Our results suggest that this positive effect may increase with expected warming and anticipated snowmelt, which may boost primary productivity where nutrients available to vegetation are rising due to the presence of geese. Undoubtedly, detailed studies taking account of vegetation type^27,78^, which was beyond the scope of the present research, will further clarify the local effects of geese-plant interactions in a context of climate change. In addition, future research on nutrient cycling in Arctic lake ecosystems will benefit from the inclusion of other food web components, given that the interactions described in this study do not work in isolation from other species in the food web.

Soil and terrestrial vegetation had lower δ^13^C values than aquatic vegetation, and sediment δ^13^C was not related to goose dropping abundance. Thus, lower δ^13^C values in sediment can be considered indicative of terrestrial inputs^36,38,83^. Accordingly, our results suggest that the allochthonous (i.e. terrestrial) vs. autochthonous (i.e. aquatic) origin of C in lake sediment was mainly driven by openness, i.e. the ratio of the lake’s surface area to that of the catchment area. The lower this ratio, the higher the relative importance of terrestrial inputs.

Runoff promotes nutrient cycling at the terrestrial-aquatic interface. In the Arctic, increased precipitation associated with climate change is likely to increase terrestrial C loading in receiving waters^3,17,21^, with effects on food webs. Indeed, seasonal alternation between terrestrial inputs and aquatic primary productivity modulates the structure and functioning of microbial communities in Arctic lakes^84^. Similarly, terrestrial input affects resource use and C transfer along food chains by zooplankton and benthic invertebrates^57,83,85^, as well as interspecific interactions in shallow lake-dwelling fish communities^86–88^. Hence, our data suggest that by modulating terrestrial C inputs, the size of lakes will mediate the effect of runoff on food webs. Given the lower quality and quantity of organic matter in soil with respect to sediment, our results also suggest that increasing terrestrial inputs may affect the detritus food chain in lakes, with implications for food web structure and nutrient transfer across trophic levels.

By modifying the feeding preferences of *L. arcticus*, differences in the quality and quantity of organic matter in sediment (SOM) among lakes affected the structure of food webs. Indeed, both food chain length and the coupling of the herbivore and detritus energy pathways were related to the diet this species, which had no aquatic predators and was able to feed on multiple food sources. The properties of the sediment did not seem to affect the presence/absence of *L. arcticus* in the study lakes, while the presence of benthic vegetation, which *L. arcticus* needs in order to lay eggs^53^, was positively associated with its occurrence. This is consistent with previous research indicating that the distribution of this species across shallow Arctic lakes in the Svalbard Islands was not greatly affected by the trophic status of the water bodies^76^. In contrast, Jensen et al.^76^ found that the abundance of *L. arcticus* decreased with water conductivity, which was higher in lakes close to the coast. While we did not observe significant differences in terms of distance from the coast between lakes with and without *L. arcticus*, future research combining information on water parameters and coverage of benthic vegetation may improve our understanding of the distribution and abundance of this key species in Arctic lake food webs.

Here, SOM represented the preferred food source of *L. arcticus*, which occupied a low trophic level and specialised on this resource (causing the number of trophic links to halve with respect to the most generalist population) when quantity and quality were high. In contrast, herbivory was higher in more generalist populations, while the consumption of *D. pulex* increased when the amount of SOM was low. SOM represents a higher quality food than benthic vegetation, it is broadly available, regardless of whether *D. pulex* is also present, and feeding on it can be considered energetically less expensive than feeding on *D. pulex* in the water column. Accordingly, our results are consistent with optimal foraging theory, which predicts that consumers will increase the number of resources consumed when the availability or quality of the most profitable one decreases^31,55^. The reduction in food chain length as a consequence of increasing resource availability at lower trophic levels is also consistent with theory^56^ and observations from other latitudes^31,37,57^. Notably, foraging optimisation by invertebrates has been demonstrated in freshwater, transitional water and marine food webs in temperate regions^31,57,89^, as well as in marine Antarctic food webs^32,37,50^, suggesting that energy constraints on foraging strategies may represent a universal driver of food web structure in aquatic communities. These results may also help predict the effects of changes in basal nutrient inputs on the transfer of contaminants in Arctic lake ecosystems, which is affected by the distribution of trophic links among species and the length of food chains^90,91^.

Fossils dated to the Triassic (around 200 million years ago) demonstrate a striking morphological resemblance to current specimens of the genus *Lepidurus*^92–94^, and *L. arcticus* is considered a “living fossil” species^94^. To the best of our knowledge, this is the first case in which foraging optimisation has been observed in such organisms. This suggests that the trophic plasticity of *L. arcticus* may have ensured its persistence in the Arctic in the face of past environmental changes, and may allow this species to cope with future variations in resource availability associated with climate change. Nevertheless, observations from our study area indicated that physical constraints (i.e. increasing water temperature) may severely reduce the fitness of current specimens, thus affecting the persistence of this species in high Arctic lakes^95^, with cascade effects on the entire food web.

The mechanisms driving nutrient inputs and food web structure presented here improve our ability to predict ecosystem-level responses to climate change in the Arctic. Specifically, our data indicate that terrestrial productivity and the presence of geese represent key biotic factors that will interact with abiotic drivers (i.e. snow coverage and lake size) in determining the rate and direction of ecological change in tundra lake ecosystems. In addition, our results provide useful information for future comparisons in both the high Arctic and other polar areas. Considering the generally increasing number of migratory geese and the lower latitudes they occupy during winter, our results may also help to better understand their impact on newly colonised areas as well as their traditional wintering ecosystems^26^.

Given their extreme latitude, density, small dimensions and relatively simple food web structure, we suggest that Svalbard lake ecosystems represent effective ecological units for monitoring and predicting the effects of climate change in the Arctic tundra. The study of these systems also represents a unique opportunity to understand the relationship between nutrient inputs and food web structure in larger and more complex lake ecosystems at lower latitudes, where warming and human pressure are profoundly affecting ecological communities^96^.

## Methods

### Ethics statement

Protocols to collect animal and vegetal samples and handling procedures were agreed with the Governor of Svalbard authority, in accordance with international guidelines and to the Svalbard Environmental Act, following official permits by the Governor of Svalbard (Research In Svalbard—Permission Application—RIS 10,242, Reference number 201500310–1).

### Study area and sampling activity

The study area was located in the Brøgger Peninsula, North Spitzbergen (Svalbard Islands) (Fig. 1). The climate of the Svalbard Islands is Arctic semi-desert. Mean annual temperatures range between − 6 and − 15 °C, while a warming trend has been confirmed for this region^4,97^. Terrestrial vegetation in the area is generally sparse and mainly represented by grasses and mosses, while aquatic vegetation is mainly composed of mosses and green algae, as generally observed in the high Arctic Tundra. The migratory Barnacle goose *Branta leucopsis* dominates the avian community and commonly occupies lake shores during the brood-rearing period (July–August). *B. leucopsis* was the only goose species observed in the study catchment areas. While other avian species can be found in the Brøgger peninsula and were actually observed in the study area, they are smaller, far less abundant, and they do not permanently occupy lake catchment areas, mostly nesting near the shore or on the beach. Arctic terns (*Sterna paradisaea*), which prevalently feed in marine coastal waters, were observed preying on *L. arcticus* during this study, as reported in the past^51–53^. In addition, Arctic foxes (*Vulpes lagopus*, a predator of Barnacle geese) were observed in catchment areas. Details of the study area, including vegetation and fauna, can be found in Lakka^53^, Jensen et al.^76^ and the literature cited therein.

Sampling was conducted in the last 2 weeks of August and the first week of September 2015 in 18 fish-free shallow lakes of glacial origin (Fig. 1). All lakes were ice-free during sampling, although they freeze solid during winter. The mean linear distance between lakes was 6.3 ± 0.6 km (Fig. 1). In most cases the lake was elongated, and six sampling sites were thus distributed on two transects parallel to the longer lakeshores. To take account of spatial variation, the distance between sampling sites varied in accordance with lake size. At each sampling site, samples of sediment, benthic vegetation, phytoplankton, the zooplanktonic *D. pulex* (Cladocera) and the zoobenthic Chironomid larvae and *Lepidurus arcticus*, which were the three most abundant and biggest macroinvertebrates in the study lakes, were collected. *D. pulex* and Chironomid larvae can be preyed on by *L. arcticus* ^52,53^. In some lakes, abundant biofilm aggregates, mainly consisting of cyanobacteria, were also found and collected (Table S2).

Sediment and benthic samples were collected at a depth of 70–80 cm. Sediment samples were collected using a cylindrical manual sediment borer (5 cm in diameter, 5 cm in height), while benthic aquatic vegetation was collected by hand. In one lake close to the coast (Lake 4), abundant allochthonous deposits of marine kelp contributed to coarse particulate organic matter in sediment. Depending on their availability within each lake, a maximum of 20 specimens of *L. arcticus* (10 per transect) were collected from both the sediment and the water column, where specimens were frequently observed to swim, in some cases preying on *D. pulex*. Specimens from sediment were collected through sieves (0.5 mm mesh size), while specimens from the water column were collected with a hand net (0.5 mm mesh size). When specimens were not found in the selected sampling sites, we extended the survey to other lake areas. Nevertheless, *L. arcticus* was never found during such additional surveys.

Chironomid larvae were collected from sediment by sieving in close proximity to the points of sediment coring. *D. pulex* was collected from the water column using a hand net (0.5 mm mesh size). To collect phytoplankton, a plankton net (20 μm mesh size, 30 cm in diameter, 120 cm in length, equipped with a 1 mm mesh filter at the mouth) was dragged horizontally approximately 20 cm below the water surface along each transect. In some lakes, phytoplankton was virtually absent and it was not possible to collect enough material to perform isotopic and elemental analyses, even after repeated samplings (Table S2). Phyto- and zoo-plankton samples were conserved separately in plastic bottles filled with pre-filtered lake water and transported to the “Dirigibile Italia” Italian Arctic Base (Ny-Ålesund, Svalbard). Here, samples were filtered, plankton samples were carefully checked under a stereoscope to remove impurities and phytoplankton samples were collected on pre-combusted Wattman GF/F filters by means of a vacuum system^98^.

To characterise the water’s main physicochemical parameters, temperature, pH and oxygen concentration were measured at each sampling site using a multiparameter probe (Hanna instruments HI9829). These data are shown in Table S4. Nevertheless, the water temperature and oxygen concentration were affected by the daily weather conditions, including wind. For this reason, since the measured parameters affected neither the isotopic signatures nor the nutrient and organic matter content in lakes (Table S4), these data will not be considered further.

Soil and terrestrial vegetation in the lake catchment areas were sampled at six sites positioned 10 m from the lakeshore facing the six sampling sites in each lake. At each site, the dominant vegetation was collected by hand. After removing the vegetation layer, soil samples were collected with the manual borer used for sampling the sediment, which was carefully cleaned with distilled water after the collection of each sample. Goose droppings were randomly collected for isotopic analysis from the lake catchment areas.

Once collected, all samples were transported to the “Dirigibile Italia” Italian Arctic Base and maintained at − 20 °C before and during transportation to Italy until stable isotope analyses.

### Catchment area characterisation

#### Goose droppings

The relative amount of goose droppings (ranking from very high to absent) was evaluated along three transects parallel to the lakeshore for each lake, and it was considered a good proxy for *B. leucopsis* abundance^29,72–76^. Each transect was ten meters long and one meter wide. A semi-quantitative scale was applied, and rankings were converted to numbers for statistical analysis^29^: very high = 4, high = 3, medium = 2, low = 1, absent = 0. For each lake, goose dropping numbers can assume a non-integer value as the average of the three transects. Based on a preliminary survey of several study lakes, Lake 3, Lake 2, Lake 6 and Lake 10 were considered as references for the rankings 4, 3, 2, 1 respectively. While the density of goose droppings was not systematically recorded during this study, the maximum density observed around lakes assigned to rank 4 (very high) varied among 70 and 90 droppings/m^2^. At the opposite, ranking 0 indicates that no droppings were observed, while less than 1 or 2 droppings per square meter were generally counted in lakes assigned to rank 1. The number of droppings observed around lakes assigned to rank 4 is consistent with the value counted in the summer of 2015 by Jensen et al.^76^, which reported a maximum density of 94 *B. leucopisis*’s droppings/m^2^ around shallow coastal lakes in Svalbard (including some lakes in our study area).

#### Digital Elevation Models and drainage maps

Detailed Digital Elevation Models (DEMs) were constructed and used to automatically map stream channel networks and delimitate the catchment area of each lake. These topographic skeletons were used to partition the catchment area into a set of runoff sub-regions and to quantify their area, volume, terrain slope and aspect. The construction of such data metrics on a node-by-node basis (each node defined by XYZ coordinates in space within a grid) enables the construction of detailed drainage basins and associated stream links, and represents the basis for an efficient catchment area network information system. For each lake, a detailed DEM was superimposed on a thematic slope and drainage direction/intensity map in order to obtain a dynamic surface map and identify the local-scale runoff affecting each lake as a function of its position in the catchment area and the total surface area of the drainage basin^99,100^. Details on the application of this procedure to lakes and their catchment areas in the study area can be found in Calizza et al.^49^.

#### Snow coverage and NDVI

Vegetation cover was analysed by computing the Normalised Difference Vegetation Index (NDVI). This index is a reliable proxy for Arctic vegetation productivity and can be related to the vegetation’s growing period and senescence^65,70,101^. The extent of seasonal snow cover was mapped with the Normalised Difference Snow Index (NDSI). NDSI can be used to detect the Snow-Covered Area (SCA) and allows separation of snow-covered pixels from cloud-, bare soil- and vegetation-covered pixels in the image^102,103^. NDSI and NDVI were derived from Landsat 8 surface reflectance products, available on demand from the USGS (https://earthexplorer.usgs.gov/), which combine digital image processing and GIS techniques using ENVI and QGIS software packages.

We aimed to analyse one image in the middle of each month from April, before the start of summer, to August. Indeed, from the first half of September the lakes start to freeze and geese start to leave. Nevertheless, in the period April-August 2015, very few images of the study area without cloud cover were available and no usable images were available for July. Thus, images for April 7, May 13, June 15 and August 13 were selected and analysed. For each image, the NDVI and NDSI were calculated in accordance with the classic equations^104–106^:

$$\begin{aligned} {\text{NDVI }} & = \, \left( {{\text{NIR }} - {\text{ Red}}} \right) \, / \, \left( {{\text{NIR }} + {\text{ Red}}} \right) \\ {\text{NDSI }} & = \, \left( {{\text{Green }} - {\text{ SWIR}}} \right) \, / \, \left( {{\text{Green }} + {\text{ SWIR}}} \right) \\ \end{aligned}$$

where “NIR”, “Red” “Green” and “SWIR” represent the spectral reflectance values obtained respectively from the 5th (NIR), 4th (Red), 3th (Green) and 6th (SWIR) bands of the LANDSAT 8 OLI satellite sensor.

To better detect the snow-covered area within each catchment area, the NDSI image was used together with the Red band to perform a supervised classification based on a decision tree algorithm. This approach is particularly effective since the Red band emphasises the spectral behaviour of snow in the visible wavelength range, thus enhancing the detection of snow pixels^102,107^. The ratio of the SCA to the total catchment area, expressed as a fraction of snow coverage in the catchment area (FSC_C_), was then computed.

FSC_C_ was > 0.90 in all catchment areas until May (Table S1). Thus, NDVI values from June to August were considered to characterise terrestrial vegetation productivity within each catchment area during the summer season. During this period, the NDVI was calculated (1) for all the pixels in each catchment area (hereafter referred to as “mean NDVI”), (2) within a buffer area of 100 m around the lake shore (hereafter referred to as “near-lake NDVI”), and (3) in the catchment area excluding the 100 m buffer around each lake (hereafter referred to as “catchment area NDVI”). The difference between near-lake and catchment area NDVI in August (referred to as ΔNDVI) was then calculated, together with the difference between August and June near-lake NDVI values, which indicated the variation of near-lake NDVI during the summer season. This allowed us to evaluate potential local-scale effects of grazing by geese on vegetation around lakes. Indeed, due to the presence of predators, geese prevalently forage in the proximity of lakes during the brood rearing period^109^, where they can achieve high grazing pressure^24^. Accordingly, we hypothesised that the higher the abundance of geese, the lower the near-lake NDVI. Lakes 3 and 8, both in the area of Ny-Ålesund, were excluded from the calculation of ΔNDVI due to the presence of human infrastructures in their catchment areas.

### Laboratory analyses

In Italy, all samples were freeze dried before analysis. Ash free dry weight as a percentage (AFDM%) was then assessed in sediment and soil samples after muffle combustion (5 h at 500 °C)^57^. The results are reported as g of ash-free dry matter per 100 g dry weight of sample. Vegetation samples were carefully checked under a stereoscope to remove impurities. Before C and N isotopic and elemental analysis, samples were ground to a fine homogeneous powder in a ball-mill (Mini-Mill Fritsch Pulverisette 23: Fritsch Instruments. Idar-Oberstein. Germany). Before being analysed for δ^13^C, soil and sediment samples were acidified to remove carbonates by adding HCl 1 M drop by drop and re-dried at 60 °C for 72 h^38^. Non-acidified subsamples were also analysed for δ^15^N. Aliquots of 5.0 ± 0.2 mg of dry powder were used for sediment, soil, vegetation, plankton and biofilm samples, while aliquots of 2.0 ± 0.1 mg were used for invertebrates and goose droppings. The powder was placed in 3.5 × 5 mm tin cups for C and N stable isotope analysis (SIA). Each sample was analysed twice and values were averaged. C and N elemental and isotopic analyses were performed using an Elementar vario-MICRO CUBE analyser coupled with an Isoprime 100 mass spectrometer, operating as a continuous flow system. Isotopic signatures were expressed in δ units (δ^15^N; δ^13^C) as the per mil (‰) difference with respect to standards, according to the equation: δX (‰) = [(Rsample—Rstandard)/Rstandard] × 10^3^, where X is ^13^C or ^15^N and R is the corresponding ratio of heavy to light isotopes (^13^C/^12^C or ^15^N/^14^N). The reference materials used were the international Vienna Pee Dee Belemnite (PDB) standard for carbon and atmospheric nitrogen (N_2_) for nitrogen. Measurement errors were found to be typically smaller than 0.05‰ for both δ^13^C and δ^15^N.

### Diet of *Lepidurus arcticus*

Within each lake, the diet of *Lepidurus arcticus* was determined using the individual isotopic values of specimens and the mean and standard deviation of the isotopic values of potential food sources. A Bayesian mixing model, returning outputs as probability distributions for the parameters of interest, was applied using the Mix-SIAR package (implemented by R software ver. 2.15.2). The output of the model is a probability density function of plausible values for the proportion of the diet accounted for by each dietary item to the diet of each organism. A whole-organism trophic enrichment factor (TEF) between *L. arcticus* and its potential food sources of 0.4 ± 0.2‰ for δ^13^C and 2.3 ± 0.5‰ for δ^15^N was applied. These values have been broadly applied in the reconstruction of the diets of aquatic invertebrates, including polar crustaceans^32,37,106^. The degree of trophic generalism of *L. arcticus* was assessed as the number of trophic links (i.e. the number of different food sources consumed) within each food web. Also, according to our hypothesis (see Introduction), we expected that the trophic position of *L. arcticus*, (TP, measured as the number of trophic steps that separated it from primary producers) varied among study lakes. Hence, within each lake, the TP of each specimen was determined according to the equation^44^:

$${\text{TP}} = \, \left[ {\left( {\updelta ^{15} {\text{N}}_{{{\text{Lepidurus}}}} {-} \,\updelta ^{15} {\text{N}}_{{{\text{aquatic }}\,{\text{vegetation}}}} } \right)/{\text{TEF}}} \right] + 1$$

were δ^15^N_*Lepidurus*_ is the δ^15^N value of a given specimen, δ^15^N_aquatic vegetation_ is the isotopic baseline, i.e. the mean δ^15^N value of aquatic vegetation within each lake, and TEF is the trophic enrichment factor as reported above. Then, the TP of the *L. arcticus* population within each lake was calculated as the mean among the TP values of all specimens in that lake. 1 is added to the formula to force a minimum TP = 2 when *L. arcticus* lies at one trophic step (i.e. one TEF) above primary producers. *D. pulex* was not considered a reliable isotopic reference for the calculation of *L. arcitcus’s* TP because (i) it was not found in all lakes where *L. arcticus* was found, and (ii) it would have not been possible to calculate the proportional contribution of the phytoplankton and the benthic food chains (which differ in their δ^15^N baselines) to the diet of *D. pulex*, given the paucity of collectable phytoplankton material in several lakes.

### Data analysis

For each lake, openness was determined as the ratio of the surface area of the lake to that of the catchment area as a whole: the lower this ratio, the smaller the lake surface area relative to the land draining into the lake.

Linear models were applied to test for dependency among variables. When necessary, multiple regression models were used to test for multicollinearity among independent variables and select the best single predictor of each dependent variable of interest. The Akaike information criterion was then used to select the best model of fit. A permutation test (9999 permutations) was performed on linear model coefficients, and the observed correlations were considered significant if (i) they were robust to permutation tests (i.e. with a permutation-based *p* value < 0.05) and (ii) the confidence intervals on slopes (95% bootstrapped confidence intervals, N = 1999) did not include zero^111^. All the linear models shown in the results section and in Appendix 1, which includes the details of the model, meet these conditions.

PERMANOVA based on δ^13^C and δ^15^N values was used to test for differences between lakes in the isotopic composition of each kind of sample (e.g. sediment, soil, *L. arcticus*). A Mantel test considering both δ^13^C and δ^15^N values was used to assess whether the isotopic signatures of sediment and soil were related to the geographical distance between lakes. The Mantel test is a permutation test for correlation between two distance or similarity matrices which makes it possible to compare multivariate data with different similarity measures^112,113^. Here, the Euclidean distance and the geographical distance based on geographical coordinates were respectively used to quantify isotopic dissimilarity between samples and linear distance between lakes.

A t-test was used to compare lakes with and without *L. arcticus* in terms of distance from the coast, goose droppings, and the AFDM%, N%, δ^13^C and δ^15^N of sediment, while a χ^2^ test was used to test the effect of the presence/absence of aquatic vegetation on the occurrence of *L. arcticus*.

Lastly, in order to compare the isotopic, AFDM% and N% values of different lake types, the lakes were classified as “*Coastal*”, “*Lowland*” and “*Glacier*”, based on their distance from the coast and the closest glacier (Table 1 and Fig. 1). Specifically, “*Glacier*” lakes had permanent glaciers within their catchment areas. “*Coastal*” lakes were further divided into “*Muddy*” and “*Sandy*” in order to reflect potential differences in nutrient and organic matter retention between muddy and sandy substrates^77^, resulting in four lake categories. Two-way ANOVA was used to test the effect of lake category and compartment (i.e. aquatic vs. terrestrial) on isotopic signatures, AFDM% and N% in both the substrate (sediment/soil) and vegetation. Single values of all the samples collected within each lake category were used to calculate the mean values (and associated standard errors) shown in Fig. 3 and to compare lake categories.

## Supplementary Information

Supplementary Information.
