# Supplementary material for: Climate-related drivers of nutrient inputs and food web structure in shallow Arctic lake ecosystems
Source: Sci Rep. 2022 Feb 8;12:2125. doi: 10.1038/s41598-022-06136-4 (PMC8825857; doi:10.1038/s41598-022-06136-4)
Supplement: Supplementary file 1 — Supplementary Information. [file 41598_2022_6136_MOESM1_ESM.docx]

**SUPPLEMENTARY MATERIAL**

**Climate-related drivers of nutrient inputs and food web structure in shallow Arctic lake ecosystems**

Edoardo Calizza^1,2^, Rosamaria Salvatori^3^, David Rossi^4^, Vittorio Pasquali^5^, Giulio Careddu^1,2^*, Simona Sporta Caputi^1^, Deborah Maccapan^1^, Luca Santarelli^1^, Pietro Montemurro^1^, Loreto Rossi^1,2^, Maria Letizia Costantini^1,2^

^1^Department of Environmental Biology, Sapienza University of Rome, Rome, Italy

^2^CoNISMa, National Inter-University Consortium for Marine Sciences, Rome, Italy

^3^ISP-CNR, Institute of Polar Sciences, National Research Council of Italy, Monterotondo (RM), Italy

^4^IRSA-CNR, Water Research Institute - National Research Council, Monterotondo (RM), Italy

^5^Department of Psychology, Sapienza University of Rome, Rome, Italy

*Correspondence: Giulio Careddu, Department of Environmental Biology, Sapienza University of Rome, Via dei Sardi 70, 00185 Rome, Italy.

E-mail: giulio.careddu@uniroma1.it

**List of supplementary materials**

Table S1: Catchment surface area, snow coverage and NDVI

Table S2: Lakes’ geographical coordinates and occurrence of food web components

Table S3: Isotopic signatures of *Lepidurus arcticus* and its potential food sources

Table S4: Water parameters

Appendix 1: Details and statistics of linear models

Table S1. Catchment surface area (CA), mean Normalised Difference Vegetation Index (NDVI) and fraction of the catchment area covered by snow (FSC_C_) in 18 shallow Arctic lakes in North Spitzbergen, Svalbard Islands, from April to August 2015. NDVI and snow cover were derived from Landsat 8 surface reflectance products, available on demand from the USGS (<https://earthexplorer.usgs.gov/>). Due to cloud cover, no usable images were available for July.

|  |  | Mean NDVI | | | | | |  | FSC_C_ |  |
| --- | --- | --- | --- | --- | --- | --- | --- | --- | --- | --- |
| Lake | CA (m^2^) | 7 April | 13 May | 15 June | 13 August |  | 7 April | 13 May | 15 June | 13 August |
| 1 | 10407 | -0.39 | -0.05 | 0.32 | 0.33 |  | 0.97 | 0.97 | 0.00 | 0.00 |
| 2 | 368651 | -0.09 | -0.05 | 0.61 | 0.30 |  | 0.95 | 0.94 | 0.12 | 0.00 |
| 3 | 134093 | -0.09 | -0.06 | 0.26 | 0.32 |  | 0.94 | 0.93 | 0.08 | 0.00 |
| 4 | 1951238 | -0.06 | -0.04 | 0.09 | 0.18 |  | 1.00 | 1.00 | 0.10 | 0.00 |
| 5 | 89989 | -0.15 | -0.06 | 0.18 | 0.30 |  | 1.00 | 1.00 | 0.03 | 0.00 |
| 6 | 1577059 | -0.03 | -0.03 | 0.10 | 0.25 |  | 0.97 | 0.93 | 0.23 | 0.00 |
| 7 | 597524 | -0.08 | -0.05 | 0.15 | 0.30 |  | 0.94 | 0.94 | 0.23 | 0.00 |
| 8 | 30587 | -0.09 | -0.05 | 0.26 | 0.24 |  | 1.00 | 0.99 | 0.00 | 0.00 |
| 9 | 354245 | -0.10 | -0.05 | 0.07 | 0.28 |  | 0.96 | 0.96 | 0.41 | 0.00 |
| 10 | 61433 | -0.05 | -0.04 | 0.22 | 0.28 |  | 1.00 | 0.92 | 0.10 | 0.00 |
| 11 | 159710 | -0.08 | -0.05 | -0.18 | 0.35 |  | 1.00 | 1.00 | 0.16 | 0.00 |
| 12 | 45557 | -0.44 | -0.06 | -0.12 | 0.25 |  | 1.00 | 1.00 | 0.86 | 0.00 |
| 13 | 381863 | -0.57 | -0.08 | -0.13 | 0.23 |  | 1.00 | 1.00 | 0.85 | 0.00 |
| 14 | 35224 | -0.10 | -0.05 | -0.12 | 0.19 |  | 1.00 | 1.00 | 0.51 | 0.00 |
| 15 | 4240445 | -0.09 | -0.05 | -0.08 | 0.09 |  | 0.95 | 0.91 | 0.59 | 0.15 |
| 16 | 174122 | -0.13 | -0.07 | -0.14 | -0.11 |  | 1.00 | 1.00 | 0.98 | 0.35 |
| 17 | 206937 | -0.71 | -0.50 | -0.11 | 0.12 |  | 0.98 | 0.94 | 0.62 | 0.00 |
| 18 | 5350844 | -0.39 | -0.10 | -0.13 | -0.02 |  | 0.99 | 0.99 | 0.90 | 0.39 |

Table S2. Gauss-Boaga geographical coordinates (East, North) and occurrence (x) of various food web components in the study lakes. Food web components include: terrestrial vegetation, benthic vegetation, *Lepidurus arcticus* (Notostraca), *Daphnia pulex* (Cladocera), Chironomid (Chir.) larvae, phytoplankton and biofilm aggregates mainly consisting of Cyanobacteria. Soil and sediment were collected for all lakes. Lakes are numbered from 1 to 18 in accordance with increasing distance from the coast.

| Lake | East | North | Terr. Veg. | Aq. Veg. | *L. arcticus* | *D. pulex* | Chir. larvae | Phytopl. | Biofilm |
| --- | --- | --- | --- | --- | --- | --- | --- | --- | --- |
| 1 | 437798 | 8771800 | x |  |  |  | x |  |  |
| 2 | 432841 | 8765525 | x | x | x | x | x | x |  |
| 3 | 434438 | 8763400 | x | x | x | x | x | x |  |
| 4 | 423198 | 8768691 | x | x |  | x | x |  |  |
| 5 | 432729 | 8764306 | x | x | x | x | x | x |  |
| 6 | 426882 | 8757293 | x | x | x | x |  |  |  |
| 7 | 436935 | 8761929 | x |  |  | x |  |  |  |
| 8 | 434053 | 8763435 | x | x |  | x | x |  |  |
| 9 | 433094 | 8763273 | x | x | x | x | x | x | x |
| 10 | 437470 | 8767800 | x | x | x |  | x |  |  |
| 11 | 431961 | 8765358 | x | x | x | x |  |  |  |
| 12 | 434080 | 8762528 | x |  |  |  |  |  |  |
| 13 | 432914 | 8762447 | x | x | x | x |  | x |  |
| 14 | 431419 | 8764665 | x | x | x | x | x |  |  |
| 15 | 426606 | 8761040 | x | x |  |  |  |  | x |
| 16 | 436096 | 8760382 | x |  |  |  | x |  | x |
| 17 | 424913 | 8764014 | x |  |  | x | x |  |  |
| 18 | 431538 | 8762352 | x |  |  |  |  |  |  |

Table S3. Mean (± standard error) δ^13^C (‰) and δ^15^N (‰) values of *Lepidurus arcticus* and its potential food sources in the study lakes. Please note that δ^13^C and δ^15^N values of organic matter in sediment is reported in Table 1 in the main text. Missing values indicate that samples were not found. Lakes are numbered according to increasing distance from the coast and capital letters indicate lake category: M: “*muddy coastal*”; S: “*sandy coastal*”; L: “*lowland*”; G: “*glacier*”.

|  | *Lepidurus arcticus* | | Aquatic vegetation | | *Daphnia pulex* | | Chironomid larvae | | Phytoplankton | | Biofilm | |
| --- | --- | --- | --- | --- | --- | --- | --- | --- | --- | --- | --- | --- |
| Lake | δ^13^C | δ^15^N | δ^13^C | δ^15^N | δ^13^C | δ^15^N | δ^13^C | δ^15^N | δ^13^C | δ^15^N | δ^13^C | δ^15^N |
| 1-M |  |  |  |  |  |  | -19.6±0.2 | 8.6±0.2 |  |  |  |  |
| 2-S | -17.6±0.2 | 5.2±0.1 | -16.5±0.4 | 4.1±0.2 | -21.1±0.1 | 4.2±0.2 | -16.0±0.5 | 4.4±0.1 | -20.4±0.1 | 3.8±0.1 |  |  |
| 3-M | -22.9±0.1 | 9.6±0.1 | -22.9±1.6 | 6.3±0.2 | -23.9±0.1 | 7.0±0.2 | -24.1±0.1 | 6.9±0.2 | -23.5±0.1 | 5.8±0.2 |  |  |
| 4-M |  |  | -16.6±1.5 | 1.4±0.3 | -20.4±0.1 | 3.8±0.1 | -17.7±0.9 | 3.4±0.1 |  |  |  |  |
| 5-S | -23.5±0.2 | 6.3±0.2 | -22.4±0.8 | 3.1±0.1 | -25.6±0.1 | 3.7±0.2 | -22.6±0.2 | 5.2±0.1 | -21.9±0.1 | 3.4±0.1 |  |  |
| 6-S | -25.6±0.4 | 5.8±0.1 | -28.0±0.4 | 3.8±0.1 | -27.2±0.1 | 4.7±0.1 |  |  |  |  |  |  |
| 7-M |  |  |  |  | -27.9±0.1 | 6.8±0.2 |  |  |  |  |  |  |
| 8-M |  |  | -24.1±1.4 | 4.0±0.3 | -27.9±0.1 | 6.5±0.1 | -28.3±1.1 | 6.0±0.4 |  |  |  |  |
| 9-L | -21.8±0.2 | 4.6±0.1 | -28.7±0.5 | 3.0±0.3 | -23.3±0.1 | 3.6±0.1 | -21.9±0.1 | 4.1±0.1 | -23.4±0.1 | 2.5±0.2 | -8.2±1.6 | 2.2±0.9 |
| 10-L | -16.9±0.1 | 3.8±0.3 | -14.1±2.3 | 2.9±0.2 |  |  | -16.4±0.4 | 3.1±0.2 |  |  |  |  |
| 11-L | -21.9±0.2 | 3.7±0.1 | -27.3±0.1 | 2.3±0.2 | -22.4±0.4 | 3.4±0.1 |  |  |  |  |  |  |
| 12-L |  |  |  |  |  |  |  |  |  |  |  |  |
| 13-L | -23.8±0.4 | 4.3±0.1 | -28.5±0.5 | 1.7±0.1 | -29.5±0.1 | 2.7±0.1 |  |  | -24.4±0.1 | 3.7±0.2 |  |  |
| 14-L | -17.2±0.3 | 2.7±0.2 | -26.1±0.2 | -0.1±0.1 | -20.9±0.3 | 2.0±0.3 | -19.7±0.6 | 0.9±0.4 |  |  |  |  |
| 15-G |  |  | -20.0±3.1 | 0.9±0.4 |  |  |  |  |  |  | -20.7±0.2 | -2.8±1.3 |
| 16-G |  |  |  |  |  |  | -28.6±0.1 | 0.3±0.2 |  |  | -13.8±0.1 | 0.2±0.2 |
| 17-G |  |  |  |  | -33.8±0.1 | 1.0±0.1 | -14.8±0.1 | 1.4±0.1 |  |  |  |  |
| 18-G |  |  |  |  |  |  |  |  |  |  |  |  |

Table S4. **a**: Mean (± standard error) temperature (T), pH and Oxygen concentration in shallow Arctic lakes in North Spitzbergen (Svalbard Islands). In Lake 13, data were not recorded due to instrument malfunctioning. δ^13^C and δ^15^N: C and N stable isotope values respectively. AFDM% and N%: ash free dry matter and N content in lake sediment respectively. **b**: regression coefficients and p values of linear models (R^2^; p uncorrelated) used to test the correlation among δ^13^C, δ^15^N, AFDM% and N% as dependent variables and T, pH and Oxygen concentration as independent variables. R^2^ = 0.01 indicates a value of 0.01 or lower. No significant effects were found (i.e. p value always > 0.05).

| **a** Lake | T (°C) | pH | Oxygen (mg/l) |
| --- | --- | --- | --- |
| 1 | 7.27 ± 0.05 | 9.71 ± 0.03 | 24.45 ± 0.06 |
| 2 | 5.18 ± 0.05 | 8.07 ± 0.01 | 22.3 ± 0.09 |
| 3 | 8.45 ± 0.06 | 7.34 ± 0.07 | 19.5 ± 0.1 |
| 4 | 5.17 ± 0.07 | 7.22 ± 0.02 | 22.83 ± 0.07 |
| 5 | 5.87 ± 0.1 | 8.09 ± 0.02 | 22.03 ± 0.18 |
| 6 | 5.98 ± 0.02 | 8.01 ± 0.02 | 22.18 ± 0.10 |
| 7 | 9.87 ± 0.03 | 8.3 ± 0.06 | 19.33 ± 0.18 |
| 8 | 2.37 ± 0.20 | 8.23 ± 0.02 | 30.83 ± 0.33 |
| 9 | 9.2 ± 0.02 | 8.28 ± 0.15 | 22.37 ± 0.03 |
| 10 | 8.67 ± 0.18 | 9.17 ± 0.01 | 21.9 ± 0.25 |
| 11 | 7.25 ± 0.03 | 8.40 ± 0.02 | 24.38 ± 0.09 |
| 12 | 8.17 ± 0.04 | 7.24 ± 0.09 | 19.78 ± 0.09 |
| 13 | - | - | - |
| 14 | 6.73 ± 0.10 | 7.33 ± 0.01 | 22.25 ± 0.27 |
| 15 | 5.52 ± 0.07 | 7.01 ± 0.02 | 22.08 ± 0.1 |
| 16 | 5.1 ± 0.06 | 7.39 ± 0.25 | 20.87 ± 0.09 |
| 17 | 6.52 ± 0.03 | 7.88 ± 0.02 | 20.83 ± 0.03 |
| 18 | 5.18 ± 0.35 | 8.23 ± 0.02 | 22.13 ± 0.24 |
| **b**  δ^13^C (‰) | 0.01 ; 0.66 | 0.01 ; 0.88 | 0.02 ; 0.61 |
| δ^15^N (‰) | 0.01 ; 0.76 | 0.07 ; 0.30 | 0.03 ; 0.50 |
| AFDM (%) | 0.01 ; 0.71 | 0.01 ; 0.77 | 0.09 ; 0.24 |
| N (%) | 0.04 ; 0.45 | 0.03 ; 0.48 | 0.04 ; 0.43 |

**Appendix 1**

Details and statistics of linear models shown in the results section. “s.e.”: standard error. “95 % C.I.”: 95 % bootstrapped confidence interval (N = 1999). “Permut. P”: permutation-based p value obtained through a permutation test (9999 permutations) run on linear model coefficients. “FSC_C_ June”: Fraction of Snow Cover in the catchment area in June. “ΔNDVI”: difference between near-lake (N.L.) and catchment area NDVI in August (see methods). “[August – June]”: variation of near-lake NDVI between August and June (see methods). “aq.” and “ter.”: aquatic and terrestrial respectively. “AFDM%” and “N%”: ash free dry matter and N content respectively. “δ^15^N” (‰) and “δ^13^C” (‰): N and C stable isotope values respectively. “L/C”: ratio of lake surface area to whole catchment surface area. Models are ordered in accordance with their appearance in the main text.

| **Independent** | **Dependent** | **slope** | **s.e. slope** | **intercept** | **s.e. intercept** | **95 % C.I. slope** | **95 % C.I. intercept** | **t** | **r2** | **p (uncor.)** | **permut. P** |
| --- | --- | --- | --- | --- | --- | --- | --- | --- | --- | --- | --- |
| Distance | FSC_C_ June | 0,00040 | 0,00007 | 0,08085 | 0,06817 | 0,00027 ; 0,00049 | -0,03111 ; 0,17222 | 5,965 | 0,690 | 0,00002 | 0,0003 |
| FSC_C_ June | mean NDVI summer | -0,36166 | 0,05975 | 0,27889 | 0,03015 | -0,46747 ; -0.24420 | 0,21113 ; 0,33879 | 6,053 | 0,696 | 0,00002 | 0,0001 |
| mean NDVI summer | goose droppings | 7,62260 | 1,19600 | 0,80822 | 0,24346 | 4,76320 ; 9,65510 | 0,53575 ; 1,11870 | 6,374 | 0,717 | 0,00001 | 0,0001 |
| goose droppings | ΔNDVI August | -0,09274 | 0,01599 | 0,13201 | 0,03206 | -0,13623 ; -0,05889 | 0,06448 ; 0,19326 | 5,800 | 0,706 | 0,00005 | 0,0001 |
| goose droppings | [August - June] N.L. NDVI | -0,11960 | 0,04241 | 0,26990 | 0,09765 | -0,17795 ; -0,02336 | 0,07988 ; 0,40224 | 2,820 | 0,332 | 0,01233 | 0,0106 |
| N.L. NDVI August | Log AFDM% sediment | 2,30180 | 0,50505 | 0,33699 | 0,11958 | 1,11960 ; 2,99300 | 0,11849 ; 0,65862 | 4,558 | 0,565 | 0,00032 | 0,0005 |
| N.L. NDVI August | Log AFDM% soil | 1,95570 | 0,42610 | 0,16927 | 0,10088 | 0,80435 ; 2,96230 | -0,04629 ; 0,41165 | 4,590 | 0,568 | 0,00030 | 0,0006 |
| N.L. NDVI August | N% sediment | 1,27330 | 0,45301 | 0,08796 | 0,10726 | 0,47950 ; 1,96330 | -0,08422 ; 0,30233 | 2,811 | 0,331 | 0,01256 | 0,0116 |
| N.L. NDVI August | N% soil | 0,87308 | 0,22599 | 0,03329 | 0,05351 | 0,24439 ; 1,41580 | -0,05008 ; 0,15629 | 3,863 | 0,483 | 0,00138 | 0,0013 |
| goose droppings | δ^15^N sediment | 1,04090 | 0,20822 | 1,10930 | 0,47937 | 0,50515 ; 1,50910 | 0,26906 ; 1,99560 | 4,999 | 0,610 | 0,00013 | 0,0001 |
| goose droppings | δ^15^N soil | 0,53370 | 0,18044 | 1,45000 | 0,41544 | 0,08997 ; 0,96482 | 0,48474 ; 2,44590 | 2,958 | 0,353 | 0,00926 | 0,0054 |
| goose droppings | δ^15^N aq. vegetation | 1,16630 | 0,30567 | 0,19360 | 0,75761 | 0,52861, 1,77380 | -1,26180 ; 1,73810 | 3,816 | 0,593 | 0,00340 | 0,0024 |
| goose droppings | δ^15^N ter. vegetation | 1,27070 | 0,33292 | -2,72790 | 0,76649 | 0,41102 ; 2,17380 | -4,21430 ; -1,04480 | 3,817 | 0,477 | 0,00152 | 0,0016 |
| goose droppings | δ^15^N *Lepidurus arcticus* | 1,64620 | 0,35037 | 1,49290 | 0,84799 | 0,98864 ; 2,58740 | -0,28698 ; 2,82620 | 4,698 | 0,759 | 0,00221 | 0,0006 |
| goose droppings | δ^15^N *Daphnia pulex* | 1,17240 | 0,31330 | 1,52270 | 0,78341 | 0,80377 ; 1,80810 | -0,12451 ; 2,44680 | 3,742 | 0,583 | 0,00383 | 0,0051 |
| goose droppings | δ^15^N Chironomid larvae | 1,49320 | 0,23411 | 0,70513 | 0,62678 | 1,03650 ; 1,94310 | -0,41268 ; 2,02240 | 6,3783 | 0,819 | 0,00013 | 0,0001 |
| goose droppings | Log δ^15^N phytoplankton | 0,11379 | 0,02792 | 0,23238 | 0,08127 | -0,06820 ; 0,16234 | 0,14593 ; 0,88472 | 4,0757 | 0,847 | 0,02666 | 0,0161 |
| δ^15^N ter. vegetation | N% ter. vegetation | 0,10063 | 0,017858 | 1,45560 | 0,043384 | 0,04031 ; 0,13414 | 1,36450 ; 1,54720 | 5,6353 | 0,665 | 0,00004 | 0,0001 |
| L/C | δ^13^C sediment* | 17,417 | 4,9932 | -25,16700 | 0,69574 | 8,22850 ; 30,1960 | -26,3620 ; -23,8170 | 3,4881 | 0,448 | 0,00330 | 0,0040 |

* Lake 4 was excluded from this model due to abundant deposits of marine Kelp, which has a similar (or higher) δ^13^C value to freshwater vegetation (Buchholz et al., 2019) and may thus confound the isotopic signal of terrestrial vs. freshwater input in lake sediment.
